# IoT-Based Medical Image Monitoring System Using HL7 in a Hospital Database

**DOI:** 10.3390/healthcare11010139

**Published:** 2023-01-01

**Authors:** Md. Harun-Ar-Rashid, Oindrila Chowdhury, Muhammad Minoar Hossain, Mohammad Motiur Rahman, Ghulam Muhammad, Salman A. AlQahtani, Mubarak Alrashoud, Abdulsalam Yassine, M. Shamim Hossain

**Affiliations:** 1 Department of Computer Science and Engineering, Mawlana Bhashani Science and Technology University, Tangail 1902, Bangladesh; 2 Faculty Member, Department of Computer Science and Engineering, Uttara University, Dhaka 1230, Bangladesh; 3 Department of Computer Science and Engineering, American International University-Bangladesh (AIUB), Dhaka 1229, Bangladesh; 4 Department of Computer Engineering, College of Computer and Information Sciences, King Saud University, Riyadh 11543, Saudi Arabia; 5 Department of Software Engineering, College of Computer and Information Sciences, King Saud University, Riyadh 11543, Saudi Arabia; 6 Department of Software Engineering, Lakehead University, 955 Oliver Road, Thunder Bay, ON P7B 5E1, Canada

**Keywords:** Internet of Things (IoT), ULSM (Ultrasound Machine), RSPI3 (Raspberry Pi 3), Health Level Seven (HL7), Minimal Low Layer Protocol (MLLP), Message Queuing Telemetry Transport (MQTT), Globally Unique Identifier (GUID)

## Abstract

In recent years, the healthcare system, along with the technology that surrounds it, has become a sector in much need of development. It has already improved in a wide range of areas thanks to significant and continuous research into the practical implications of biomedical and telemedicine studies. To ensure the continuing technological improvement of hospitals, physicians now also must properly maintain and manage large volumes of patient data. Transferring large amounts of data such as images to IoT servers based on machine-to-machine communication is difficult and time consuming over MQTT and MLLP protocols, and since IoT brokers only handle a limited number of bytes of data, such protocols can only transfer patient information and other text data. It is more difficult to handle the monitoring of ultrasound, MRI, or CT image data via IoT. To address this problem, this study proposes a model in which the system displays images as well as patient data on an IoT dashboard. A Raspberry Pi processes HL7 messages received from medical devices like an ultrasound machine (ULSM) and extracts only the image data for transfer to an FTP server. The Raspberry Pi 3 (RSPI3) forwards the patient information along with a unique encrypted image data link from the FTP server to the IoT server. We have implemented an authentic and NS3-based simulation environment to monitor real-time ultrasound image data on the IoT server and have analyzed the system performance, which has been impressive. This method will enrich the telemedicine facilities both for patients and physicians by assisting with overall monitoring of data.

## 1. Introduction

Since the Internet of Things (IoT) has a significant role in a variety of areas, including IoT-based healthcare, intelligent buildings, and smart monitoring [1], the evolution of smart medical sensors, gadgets, cloud computing, and healthcare technology is attracting major interest from academics and the healthcare business. With the introduction of real-time analytics in healthcare, we see technology exceeding the limits of what we can currently achieve in this sector. IoT has been recognized as being one of the most significant research topics in the field of medicine, particularly in image processing. Furthermore, patient engagement and satisfaction have also been enhanced, and interactions with doctors have become easier and more efficient. Remote monitoring of a patient’s health has also shown to lead to a reduction in hospitalizations and re-admissions. IoT is now also having a huge impact on medical costs and clinical outcomes. However, medical devices have now evolved to the point where a large amount of data are generated in the healthcare environment daily. The advancement of medical technologies such as magnetic resonance imaging (MRI), computed tomography (CT), and ultrasound imaging generates vast amounts of data on a daily basis, and the data collected from these devices include multiple dimensions and factors [2]. The dimensionality of medical image databases is rising exponentially, making it difficult to manage file systems because of the rising amount of data stored due to the growth of medical databases, so the handling of medical data has become a top priority for healthcare providers [3].

A few research projects have created a method for transmitting real-time ultrasound data via the internet. A smart handset or computer displays the ultrasound image as well as other ultrasound data. For detecting kidney abnormalities, a portable ultrasound imaging system based on FPGA and IoT is also being developed [4]. A study which presents an algorithm for detecting and classifying renal abnormalities, uses LUT (Look Up Tables) and SVM (Support Vector Machines) with MLP (Multi-Layer Perceptron) based on an IoT e-health architecture [5]. Another piece of research proposes an electronic healthcare method based on IoT. It has now become possible to transfer real-time ultrasound video over the internet [6]. Another study has provided three types of modifications: a modified multipath routing protocol, an NS3-program used to simulate a real-world network to improve the modification, and a system that included the implementation of some algorithms such as EIGRP (Enhanced Interior Gateway Routing Protocol). The goal is to gather information about nearby nodes and add it to the routing table, then determine the best routes while keeping the cost of the value obtained as guide information from the router in mind. The protocol routing tables are regularly updated, resulting in topological changes and information being updated from one router to another, resulting in additional convergence [7]. Yet another study reviewed how to construct a new model in NS3, where they utilized a wireless scenario for MANETs (Mobile Ad-hoc Networks) with different parameters to analyze the behavior, and they found that the YansErrorModel had a greater success rate in most scenarios [8]. The goal of a further piece of research was to see if it was possible to transfer pictures of ultrasound imaging in rural/undeveloped settings. Aiming to benefit telemedicine, they developed an Arduino-based ultrasonic developer kit [9].

Previous research states that ultrasound images are essential for medical purposes and for physicians. As a result, it is critical that ultrasound images can be observed, and even better if physicians can do this from remote locations with limited bandwidth. Monitoring from remote locations is necessary, but it would be ideal to build up a real-time visualization dashboard for all clinicians at any institution. This would bring data sources into closer proximity to the healthcare facilities where they are analyzed [10]. The advancements in fog computing, cloud computing, and IoT technologies are improving the healthcare industry and might save billions of lives by enabling quicker public access to facilities and services. Another report proposes an intelligent real-time patient monitoring system with pinpoint accuracy that detects any irregularity in the subject’s vital data, such as temperature, and includes a fall detection model [11]. Their main objective is to design and construct a mobile IoT-based healthcare system that gathers patient data from different sensors and promptly notifies both the doctor and the patient’s guardian via emails and SMS. It analyzes the patient’s vital data remotely and detects ailments quickly [12]. Additionally, an IoT-based human health monitoring system has also been established which measures the user’s blood pressure, pulse, body temperature, heart rate, physiological information, and other data about a patient’s vital signs. After collecting long-term data, factors associated with a probable risk prediction should be investigated further in the future to expand the use of health monitoring systems [13]. In the future, this will provide a scientific and practical foundation for the prevention and treatment of chronic high-risk illnesses. Another study suggests a smart city health monitoring system. The proposed technique calculated a spectrum by using LPCC (Licensed Professional Clinical Counselors) to discriminate between individuals with normal and disturbed speech and detect the reason for this. Running speech is used to test the system’s ability to grasp people’s ordinary conversations and detect the existence of voice anomalies [14]. Perhaps this might be utilized in clinics to provide medical practitioners with extra information on the occurrence of voice anomalies. The recommended technology could also be placed on a soldier’s body and use the Global Positioning System to track their health and current state. These data would be sent to the control room via the Internet of Things. The Gustier detection system, which employs emergency signaling sensors, would also be part of the envisioned system.

### Related Works

Therefore, a model has been proposed that might be utilized to implement a low-cost strategy for the protection of human life on the battlefield [15]. Another study shows how IoT significantly affects the healthcare system [16]. A solution in another study describes a patient wearing a single wristband to capture vital sign data, decreasing the number of sensors required per individual to just one [17]. The study provides a schematic representation for a low-cost IoT-based biomedical kit that can monitor vital signs for persons in rural areas at a rapid rate with an accuracy of 98% and 95% for temperature readings and pulse rate, respectively. In a further study, Musical Chairs is applied to two well-known image recognition models, AlexNet and VGG16, and is implemented on a Raspberry Pi network. In terms of time and energy performance, their systems were compared to the Tegra TX2, an embedded low-power device featuring a six-core CPU and a GPU. This configuration demonstrates how Musical Chairs enables the collaboration of IoT devices to achieve equal real-time performance without spending the additional costs of running a server [18]. Overall, our approach proposes helping patients deal with challenging situations while doctors are absent. It does not help in emergency cases where patients must be admitted to hospitals, but it does help in giving appropriate first aid protocols for dealing with emergencies. Most people who are suffering from common symptoms of an illness can benefit from this idea.

Medical images are typically disseminated to numerous organizations or within organizations for correct diagnosis. Therefore, the exchange of medical data is required to improve the quality of healthcare services. Users will be able to employ accessible and distributed cloud computing platforms, and so IoT device connections through the internet and advancements in cloud computing technology are required. The ability to watch real-time picture data from an IoT server simplifies the process for both patients and clinicians. However, due to low data transmission rates and payload size, dependable and real-time picture transmission in IoT monitoring systems is regarded as very difficult. IoT protocols are message-centric wire protocols developed for M2M communications that allow the transfer of telemetry-style data in the form of messages from devices to a server or small message brokerage over high latency or limited networks. MQTT, MLLP and CoAP meet these requirements by utilizing tiny message sizes, message management, and lightweight message overhead. Real-time picture data monitoring systems using MQTT and MLLP protocols are difficult for IoT servers to handle. This study proposes a strategy to remedy this problem.

The main contributions of this study are given below:

1. Machine-to-machine-based communication with an IoT server using HL7 message processing.

2. Medical diagnoses image data stored in an FTP server with unique encrypted image links.

3. A real-time image data display with patient info in an IoT server.

The rest of the paper is organized as follows: (i) proposed methodology, with discussion on how to transfer telemetry data with images; (ii) results analysis; (iii) discussion, and (iv) conclusions.

## 2. Materials and Methods

Machine-to-machine-based data communication and monitoring of patient diagnostic information over IoT servers is difficult and time consuming. To overcome the above problem, this study proposes a new method where the following protocols and methods are used to improve accuracy: MLLP protocol, HL7 messaging, FTP based storage, RSA based encryption and decryption, and MQTT protocol. This study uses HL7 messaging for machine-to-machine-based communication, and for large data storage uses an FTP server. The MQTT protocol is used to communicate with an IoT server using RSA-based encryption and decryption.

In this approach, our proposed model divides the entire procedure into four parts, which are as follows:

- Ultrasound Machine to Raspberry Pi HL7 messaging process;
- Result data stored in LIS (Lab Information System);
- Raspberry Pi to FTP server image transfer;
- Transfer of files from FTP server to MLLP and MQTT protocol with the help of a Raspberry Pi 3 (RSPI3).

**Figure 1:**
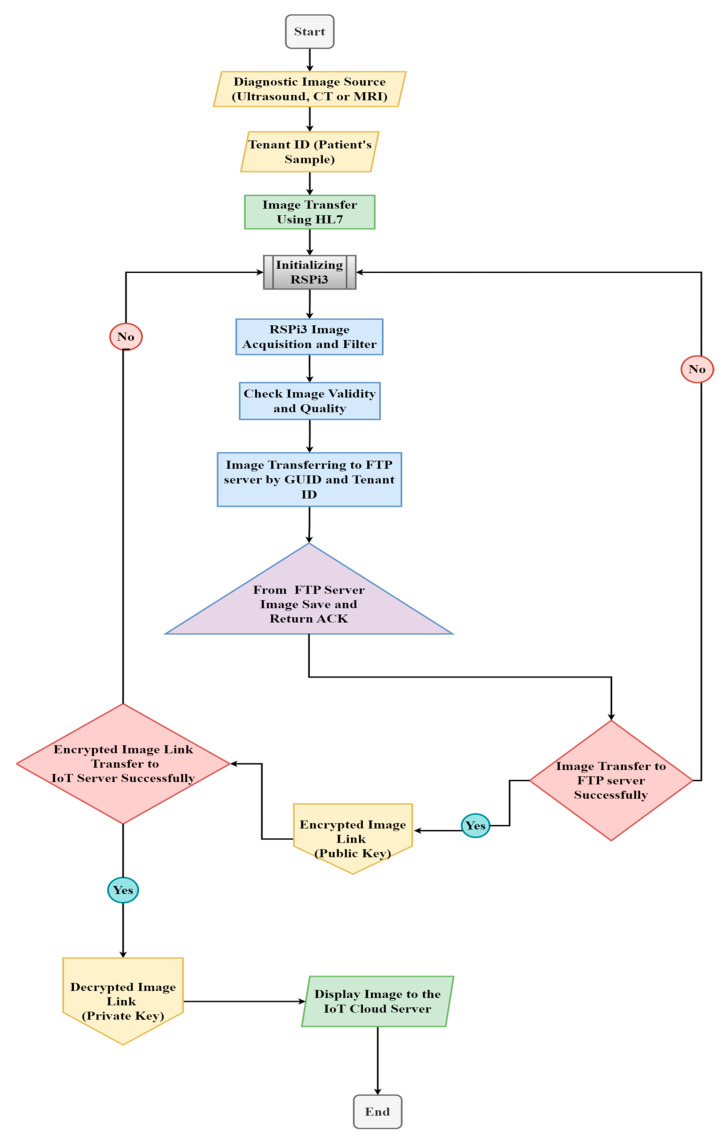
Flow chart of the whole process.

The above steps are displayed as a flowchart in Figure 1.

This study obtained a variety of options from the analysis for using database platforms to track the health status of patients. Comprising observations and research procedures, this study provides solutions for monitoring patient data. From looking at previous studies, this study also found several bottlenecks when monitoring image data in real time in hospital databases.

**Figure 2:**
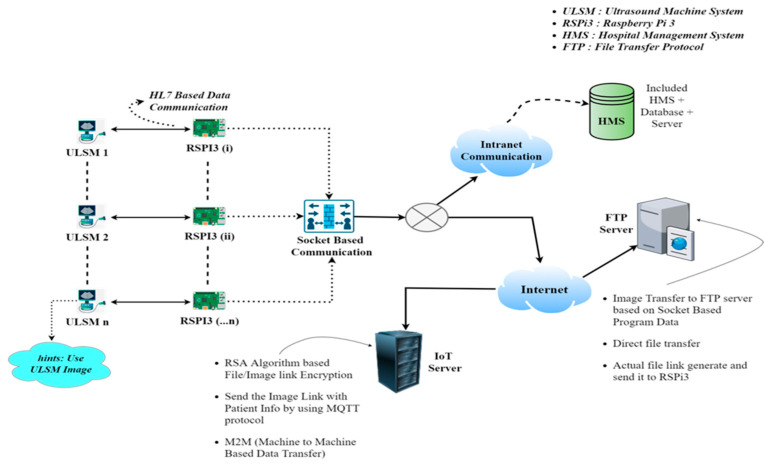
Schematic diagram (full system).

Before being sent to cloud storage, a microprocessor processes all of the output raw data. With the use of IoT, physicians can simply view that information. This solution heavily relies on the Raspberry Pi for real-time data processing and the IoT server i.e., thingsboard.io, an open-source IoT platform [19]. However, the main issue of transmitting image data directly to the IoT server via the MQTT and MLLP protocols is handled in our provided approach with a new methodology which overcomes the image viewing problem in real time. A custom widget in the IoT server also aids in the deployment of a machine-to-machine based custom dashboard with image and physician-based access. The overall schematic diagram is shown in Figure 2.

However, physicians may not need to consider latency, suitable bandwidth management, or fog computing, or to wait for clearance from others for precise therapy and prescriptions, if the diagnosed ultrasound images are processed and transformed with HL7 in such a way that only uses the patient’s sample ID for the entire communication. Converted ultrasound images of patient diagnoses will be stored in hospital databases, and precise ultrasound image conversion and sharing links to the images in an IoT server will ensure precision monitoring of patient illnesses, and thus patients will receive appropriate treatment from doctors at any time. The proposed model directly acquires data from ultrasound equipment. The video is converted to photos using the most up-to-date ultrasound machine, which is ideal for the experimental results. The transformed image is sent to RSPI3 in HL7 base64 format, along with patient data. RSPI3 processes the image and converts it into a jpg or png file, which will then be delivered to the FTP server. Again, socket-based (IP + PORT) communication connects the ultrasound machine to the RSPI3. One side is the listener, while the other is the sender, or vice versa. In this instance, the proposed method uses an HL7-based data transfer method. Ultrasound machines often convert ultrasound raw data to video exclusively. Physicians extract visuals from the video that are ideal for visualizing patient information. This image is transformed to base64 format before being sent to the LIS computer. An RSPI3 was used as the LIS in this case. This RSPI3 takes an HL7 message and converts the base64 image to JPG or PNG format using the HL7 messaging protocol. The image quality is also checked by the RSPI3. The patient sample ID is sent in an HL7 message with each image data (see Section 2.1.1).

To assign and show the patient information through a machine-to-machine dashboard on an IoT server, the proposed method utilizes thingsboard.io in this case [19]. Here, our primary research experiment is to set up monitoring of image data collected from hospital machines using IoT servers, with the process going via Raspberry Pi to the IoT server. The proposed model uses ultrasound equipment for this purpose, which will gather ultrasound images from patients and monitor the image data from the IoT server without transferring the image files. The core benefit here is that we can easily monitor image data from an IoT server without sending image files in real time. The proposed model also transfers telemetry data and an image-encrypted link to the IoT server.

### 2.1. Ultrasound Machine to Raspberry Pi HL7 Messaging Process

Ultrasound technology has advanced dramatically in the last decade, enabling tremendous downsizing in ultrasound equipment design and manufacture. Presently, ultrasound technology ranges from small machines that fit in the palm of one’s hand to high-end equipment that can provide complicated ultrasound testing.

**Figure 3:**
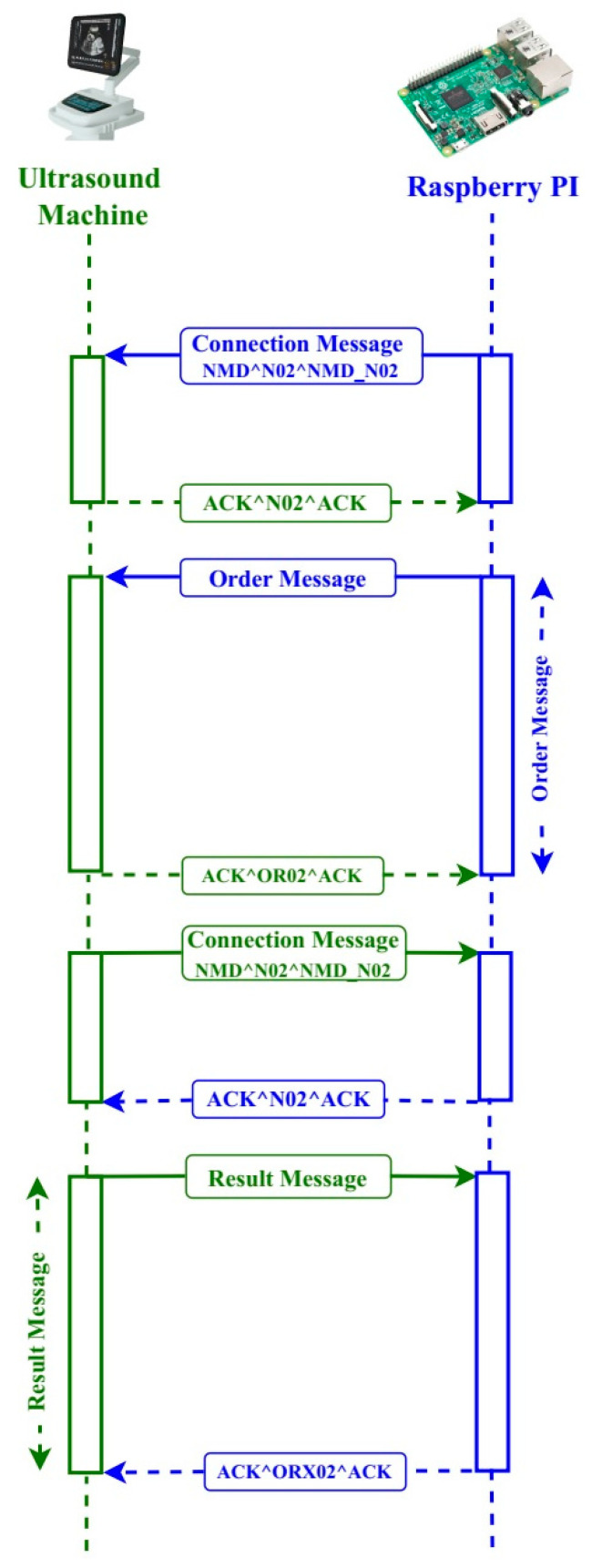
Message timing diagram from an ultrasound machine to Raspberry Pi.

HL7 messages are used to transfer data between systems and make a sequence of segments, with individual segments or groups of segments being optional, obligatory, or repeated (HL7 cardinality). The message timing diagram in Figure 3 illustrates how the ultrasound machine (ULSM) and RSPI3 interact with one another. They interact with each other on demand via intranet connection, based on order and result messages.

The ULSM communicates with the RSPI3 based on messaging protocols which use the following sequence:

(i). Connection Message
(ii). Connection Acknowledgement
(iii). Order Message
(iv). Order Acknowledgement
(v). Result Message
(vi). Result Acknowledgement

They check in with each other via a connection message, NMD^NO2^NMD_NO2, before communicating. If the connection is effective, the other component responds with an acknowledgement message, ACK^N02^ACK (Figure 3) and then the connection is made. Otherwise, it will notify the user that the other machine is down or that the connection has been broken. The RSPI3 sends an order to the ULSM based on the connected patient order with that sample ID. After acquiring the order message, the ULSM places it in a queue. An acknowledgement message, ACK^OR02^ACK, is returned.

**Figure 4:**
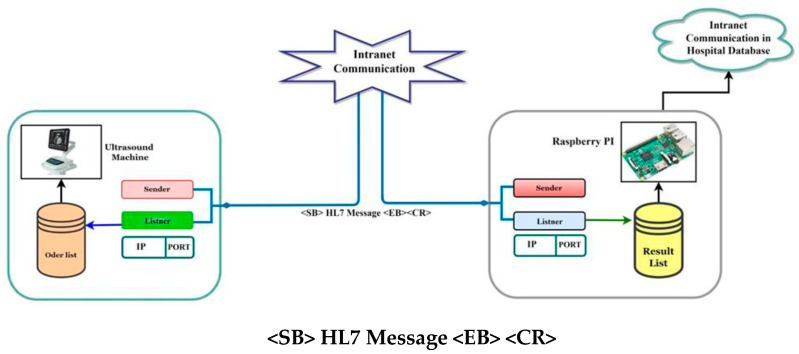
HL7 communication process, ultrasound machine to Raspberry Pi.

The same procedure is used to send the observation result message (Figure 4).

The Minimal Lower Layer Protocol (MLLP) and Socket Programming are two protocols that must be followed when transmitting HL7 (Health Level 7) messages from one end point to another. The proposed model uses socket programming processes where listener ports are open in distinct ports on both sides to accept incoming messages. Both systems are linked via intranet. RSPI3 transmits an order list from the LIS to the ULSM, which receives it and stores it in a queue on the local system. ULSM, on the other hand, sends the result to the RSPI3, which receives the list as a queue. HL7 messages are used to communicate between the two sections.

**Figure 5:**
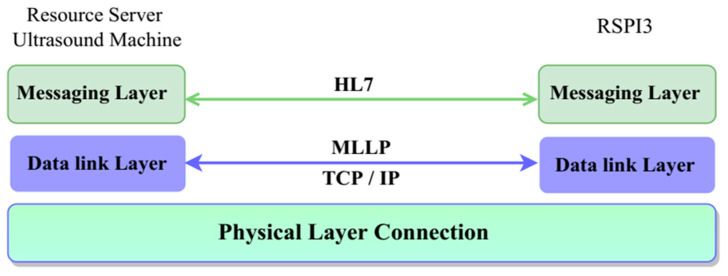
HL7 protocol layer-based connection.

The HL7 message is encoded in bytes and sent to the other side (Figure 5).

#### 2.1.1. HL7 Block Formation and Messages

The HL7 block format is as follows:

**<SB>Message<EB><CR>**

Here:

**<SB>**—SB stands for Start Block Character, which is a 1-byte ASCII character, such as

**<VT> or <0x0B>.**

**Message—**This denotes a message with a certain number of bytes of data. It is a block of HL7 data content.

**<EB>**—End Block, or EB, is a 1-byte ASCII character such as <FS> or **<0x1C>.**

**<CR>**—CB denotes a carriage return containing one byte of data. For instance, **0x0D.**

There are two types of connection from the ULSM to the RSPI3 and vice versa:

(a) Active connection and (b) Passive connection.

Here, the proposed model used an active connection –

(a)Active Connection:

Connection established on both sides **(i) Persistent (ii) Transient**

**(i) Persistent**

1. On startup, try to connect to the listening or passive system’s set IP address and port.
2. If the connection fails, wait a brief time before retrying the connection. Once all possible retries have been used up, the connection will either be deactivated at the operator’s request, or the instrument will be switched off.
3. Maintain the connection at all times, even if there are no further messages to send.
4. Retry the connection if a disconnection is detected outside of a data exchange.
5. A message transmission fault is displayed if a disconnection is detected during a data exchange (for example, while waiting for a response), and the ULSM stops the HL7 connection.

The above steps are described in Algorithms 1–3 accordingly.

Algorithm 1 shows how the ULSM communicates with the RSPi3, Algorithm 2 shows how the RSPi3 communicates with the ULSM using socket programming, and Algorithm 3 describes how the listener receives HL7 messages.
**Algorithm 1: Ultrasound Machine to RSPI3 Message Transfer****1**Start**2****while** *UltrasoundMachineWantsToSendMessage* **do****3**    **if** *is ConnectionIsActive*
**then****4**      Send HL7 Message to RSPI3 [SB EB CR]**5**    **else****6**      **while**! *isConnectionIsActive*
**do****7**        isConnectionIsActive = Established A Socket or TCP/ IP [IP + PORT] Connection With RSPI3**8**       SLEEP(Few Seconds)**9**End

**(ii) Transient**

1. Try to connect only when a message is ready to be sent.
2. When a message is ready to be sent, connect to the listening or passive system’s preset IP address and port.
3. If the connection fails, it needs to wait a few moments before attempting it again, as in the Persistent method.
4. Only keep the connection open if there are further messages to send. Close the connection if this is not the case.
5. A message transmission fault is displayed if a disconnection is detected during a data exchange (for example, while waiting for a response), and the ULSM stops the HL7 connection.

**Algorithm 2: RSPI3 to Ultrasound Machine Message Transfer**

**1**
Start
**2**
**while** *UltrasoundMachineWantsToSendMessage* **do**
**3**
    **if** *isConnectionIsActive* **then**
**4**
     Send HL7 Message to Ultrasound Machine [SB EB CR]
**5**
   **else**
**6**
     **while**! *isConnectionIsActive*
**do**
**7**
      isConnectionIsActive = Established A Socket or TCP/ IP [IP + PORT] Connection With Ultrasound Machine
**8**
        SLEEP(Few Seconds)
**9**
End


**Algorithm 3: Listener**

**1**
Start
**2**
**while** *ConnectionIsOpenForListning* **do**
**3**
     Receive HL7 Message in byte code and Convert it to Readable format
**4**
End

In HL7 communication, different kinds of messages are used, such as the Connection Messages (see Table A1), Order Messages (Table A2), Result Messages (Table A3), and Acknowledgement Messages (Table A4).

### 2.2. Result Data Extraction

The ultrasound machine sends a result message based on HL7 via a socket-based connection. The HL7 result message contains a few segments which are MSH (Message Header), PID (Patient information with ID), SID (Sample ID), OBR (Observation Result), and OBX (Observation Result Data). From those segments, the proposed model extracts that information via a segment-based data extraction model, which is a string-based concatenation model.

After that, the extracted result data are used for further processes such as (i) storing in the database, or (ii) data processing for FTP and IoT.

#### Result Data Stored in the LIS (Lab Information System)

(a) Raw data of the results are stored in the LIS (Laboratory Information System) via API (Application Programming Interface).
(b) Extracted data are:
  (i). Patient’s info
  (ii). Sample ID
  (iii). Extracted Result data stored in the database.

After getting the result message from the ULSM (Ultrasound Machine), the proposed model categorizes the message into two parts: (i) saved raw result data (ii) extracted result data. Here, the proposed model worked with (ii) the extracted result data.

Here, real-time data mean raw formatted data which have been sent by ULSM and are stored in LIS storage, and thereafter any changes will be strictly prohibited.

**Figure 6:**
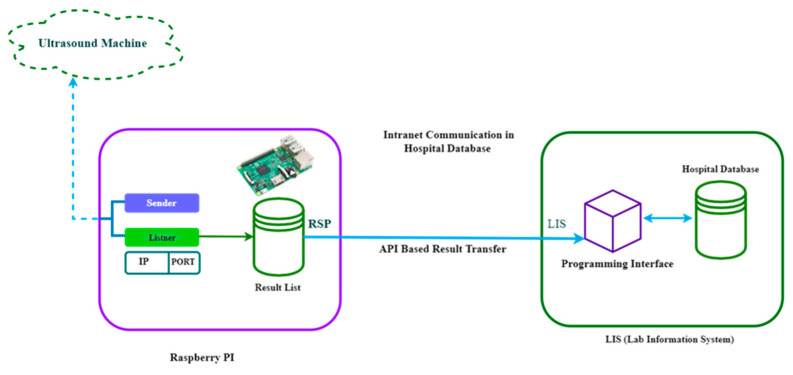
RSPI3 to LIS result data transfer.

The result data are transferred from the RSPI3 to the LIS via the API over the intranet connection (Figure 6). Here, messages are stored in the main database every time the validity of upcoming messages are checked in LIS based on the API token. Therefore, no unwanted message will be stored in the LIS because of the API token. The raw result data are stored in the database as a response. Extracted data from result messages are also recorded in the database using the patient record ID and sample ID, which is then utilized for further processing.

### 2.3. Raspberry Pi to FTP Server Image Transfer

**Figure 7:**
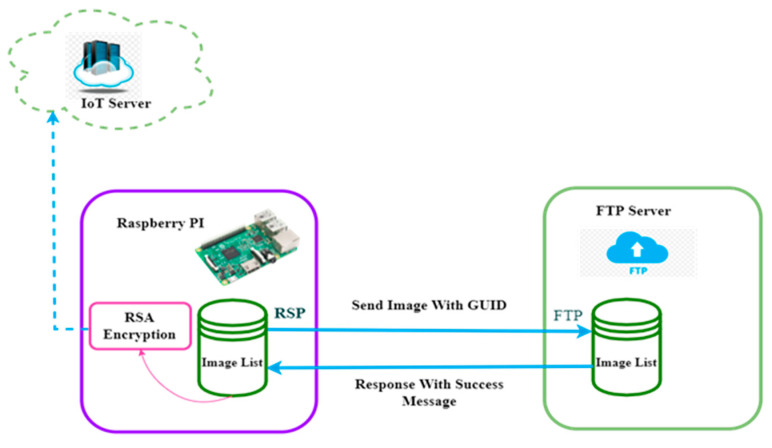
Image transfer process from RSPI3 to FTP.

After getting a message from the ultrasound source and storing data in the LIS, the Raspberry Pi processes the HL7 messages and finds out patient information, sample ID, and doctor information using the Base64 image. The Base64 image is transferred to the FTP server database as a formatted image with a Globally Unique Identifier (GUID). The image is renamed with the GUID and sent to the FTP server using the FTP server user ID and password. After the image is successfully transferred to the FTP server, it responds with a unique image link using the GUID. This process is described in the following steps:

(i). Convert the image from Base64 to JPG, or get the image from HL7 or the direct Base64 image.
(ii). Construct the GUID (Globally Unique Identifier) for the image.
(iii). Rename this image with the GUID send it to FTP server.
(iv). From the FTP server generate a unique image link and send it back to the Raspberry Pi. After getting the response message from the FTP server the RSPI3 encrypts the image link via RSA public key and then sends the link to the IoT server (Figure 7).

Algorithm 4 is used in this process to:

(a) Generate the GUID.
(b) Transfer Base64 image data using the GUID to the FTP server.
(c) Get Base64 image links from the FTP server.
(d) Encrypt the image link via public key.
(e) Transfer the encrypted image link to the IoT server.

Those images are also mapped based on sample ID with GUID as per the following steps:

#### Encryption Process

The following RSA encryption process is carried out based on the GUID of an image. This study uses the key and messages as shown in Table A5, Table A6, Table A7, Table A8 and Table A9.

Algorithm 4 describes how the Raspberry Pi sends the image to the FTP server.
**Algorithm 4: RSPI3 to IoT Server Image Transfer**
**Input:** Image data from Ultrasound Machine **Output:** boolean:true/false**1**Start**2**IoTServerResponse = false**3**
 **while** *GetImageDataFromUltrasoundMachine* **do**
**4**     **if** *ImageDataIsValid*
**then****5**      ftpResponse = FTPServer(imageData)**6**        **if**
*ftpResponse* == *IsValidImageLink*
**then****7**imageLink = ftpResponse[‘imageLink’] encryptedImageLink = ENCRYPT(imageLink, publicKey) IoTServerResponse = TransferImageToIoTServer (encryptedImageLink)**8****return** *IoTServerResponse***9**End

### 2.4. RSPI3 to IoT Server Image Transfer

The MQTT protocol is used to send the encrypted image link to the IoT server. After producing an encrypted image link with a login and password from an FTP server, the image link is transferred to a real-time IoT server. This phase configures the real-time IoT server. This image is displayed in the IoT server alongside patient data. If a patient’s ultrasound image cannot be transmitted to an IoT server, the image is re-sent. The Raspberry Pi not only sends the encrypted image, but it also sends the patient information using the MQTT protocol. This process is described in the following steps:

(i). Get the encrypted image link from the FTP server.
(ii). Transfer the encrypted image link to the IoT server using the IoT client username and password.
(iii). Get the IoT response for image link transfer, if yes then step 4 else step 1.
(iv). Then, decrypt the image link and display it in the IoT server based on the client.

These steps are based on Algorithm 5.

Algorithm 5 describes the Raspberry Pi to IoT server encrypted link transfer process.
**Algorithm 5: RSPI3 to IoT Server Image Link Transfer**
**Input:** *encryptedImagelink*, *patientInfo encryptedImagelink* is a string and *patientInfo* finite set *patientInfo* = ***{****patientInfo*_1_*, patientInfo*_2_*, …, patientInfo_n_**}*** of JSON **Output:** boolean info true or false1Start2**while** *EncryptedImageLinkIsValid* **do**3    IoTServerResponse = IoTServer(*encryptedImagelink,patientInfo*)4    **if** *IotServerResponse* == *false*
**then**5     IoTServer(*encryptedImage,patientInfo*)6**Function** IoTServer(*encryptedImagelink,patientInfo*):7     *ecryptedImageLink* = DECRYPT(encryptedImagelink,privateKey) *isDisplayed* = displayInfo(*decryptedImageLink,patientInfo*)8**return** *isDisplayed*9**Function** displayInfo(*decryptedImageLink,patientInfo*):10     *isDisplayed ← false*11     **if** *decryptedImageLinkisanImage And patientInfo*
**then**12       Display Image to assign Dashboard in IoT Server13       *isDisplayed ← false*14    **return** *isDisplayed*15End

## 3. Results and Discussion

Data collected directly from hospital experiment machines show that ultrasound equipment is not practicable since the observed data are in a raw format and require preprocessing as well as a LIS (lab information system). However, a large number of devices send data to several patients at the same time. Managing and monitoring such operations in real time is thus more challenging and time intensive. Finally, the proposed model was able to reach an overall situation where this model could calculate data response times based on bandwidth and data packet size. The proposed model also measured the patient information sent, the data transmitted each time, and the total data sent each time for the given criterion. The result was created based on the minimum time it took to send the data to the IoT server.

**Figure 8:**
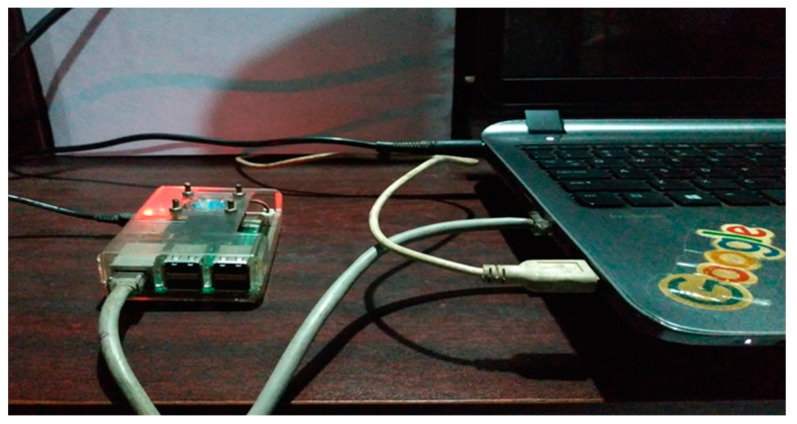
LIS to RSPI3 connection.

The LIS and RSPI3 connection is shown in Figure 8. The patient information is sent using an HL7 message after extraction. They are connected using socket programming via an ethernet cable.

**Figure 9:**
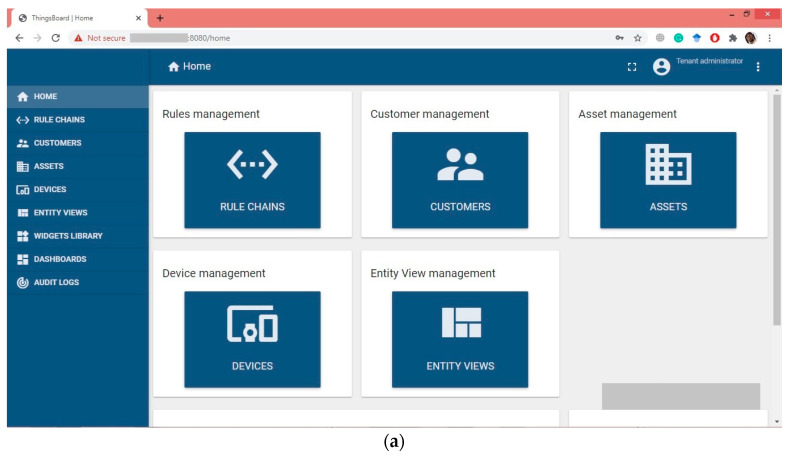

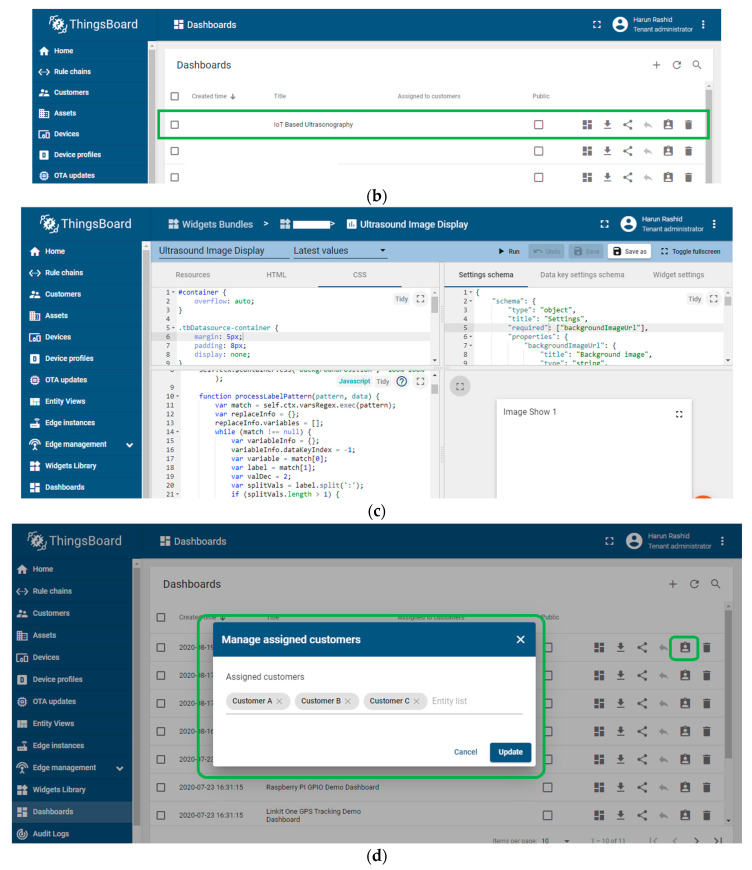
Thingsboard configuration: (**a**) Thingsboard dashboard (Main); (**b**) ultrasound machine dashboard. (**c**) Displaying images using a custom widget; (**d**) assigning user-based dashboards (access control).

Patient information results are shown in the IoT server where the proposed model uses thingsboard.io in a demo server. In this part, the proposed model needs to configure the IoT server to display that information. In Figure 9, the thingsboard configuration is shown. Figure 9a shows the possible thingsboard options for configuration and Figure 9b shows a machine-to-machine based dashboard (for the proposed model only one dashboard was used). Figure 9c shows the most important part of the image display, a custom widget which is customized for image viewing only. This widget is customized for image link decryption using the RSA private key and displays the image which is linked with the dashboard. User-based dashboard access control for the proposed model is shown in Figure 9d, where access to specific dashboards can easily be assigned to users. A summary of the overall configuration of the thingsboard is explained in Table 1.

**Figure 10:**
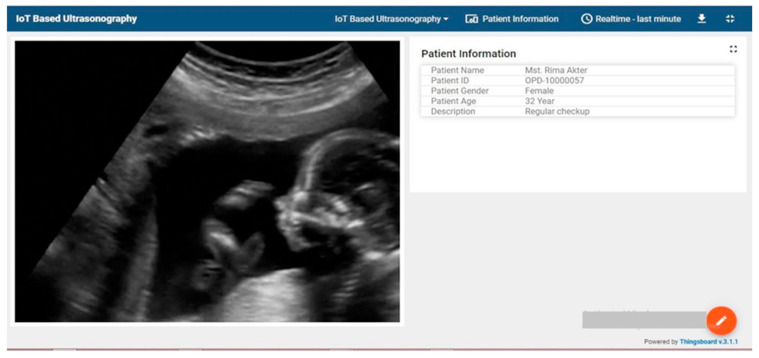
Ultrasound image view with patient information.

After configuring thingsboard with custom widgets, physicians can easily see patient information with images (Figure 10). On the left side of Figure 10, ultrasound image data are displayed. On the right site patient information is shown. This is the final output of the image and patient info which is displayed on the IoT server in real-time.

After requesting patient information from the IoT server, the proposed model examines different sized images based on different bandwidths and checks the IoT server response for displaying those images. Here, the proposed model uses 1 MB to 5 MB images which are displayed on the IoT server depending on the bandwidth. Table 2 shows that image display delay time varies based on image size as well as bandwidth. Lower bandwidth with small packet size means that it takes more time to show the information on the IoT server. On the other hand, higher bandwidth with higher packet size means it takes less time to display (Table 2).

In addition to the real-time situation, the proposed model also simulates the whole process using NS3 in two parts: (i) ULSM to hospital management system (HMS), and (ii) HMS to FTP and IoT.

NS3 allows us to trace the node pathways, which allows us to determine the number of data points sent or received. Trace files are created to track these activities. Its main purpose is to mimic networks of connecting nodes and the traffic that flows between them. To do this, NS3 offers a core abstraction of computer nodes with applications to produce traffic as well as net devices and channels to transport the traffic.

**Figure 11:**
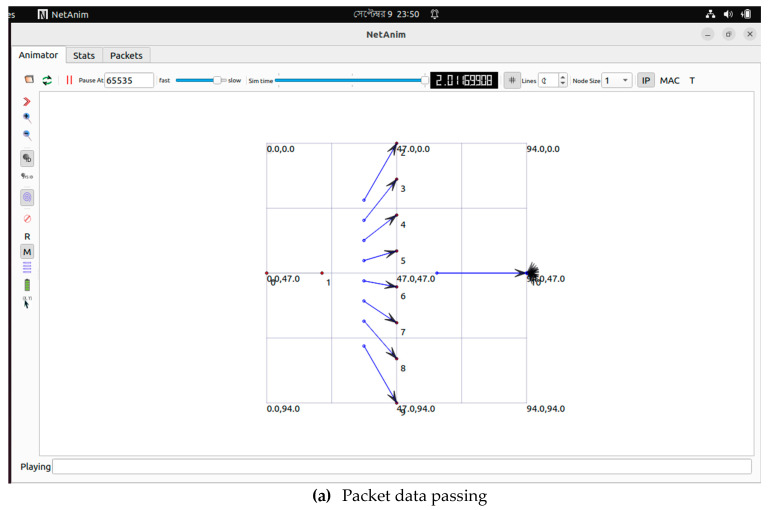

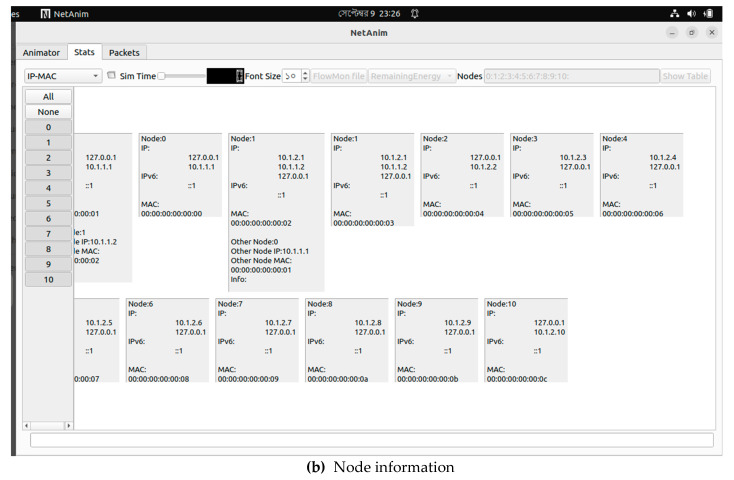
NS3 simulation from ULSM to HMS. (**a**) Packet data passing; (**b**) Node information.

In part (i) of the ULSM to HMS simulation, image data transfer employed a total of 10 nodes, each labelled with a number from 1 to 10. Node 1 is the source node from which the proposed model transfers packet data using ULSM. This study employed nodes 2 to 9 as intermediate nodes, such as the RSPI3, and Node 10 as a server, receiving packets from different nodes and responding with acknowledgement (ACK) messages (Figure 11).

**Figure 12:**
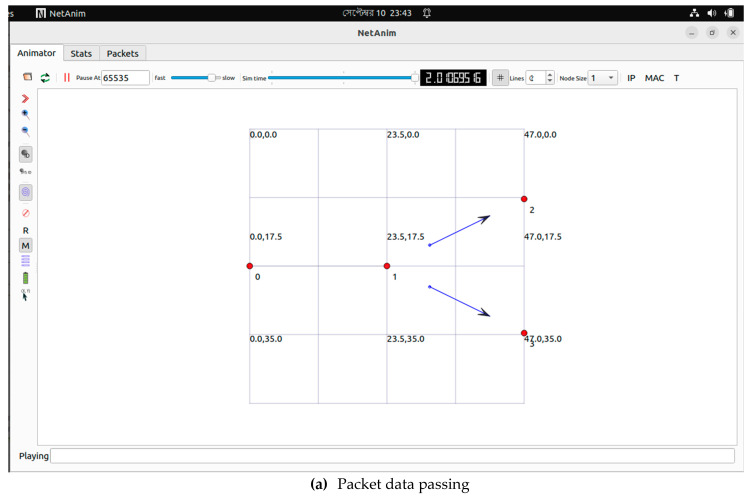

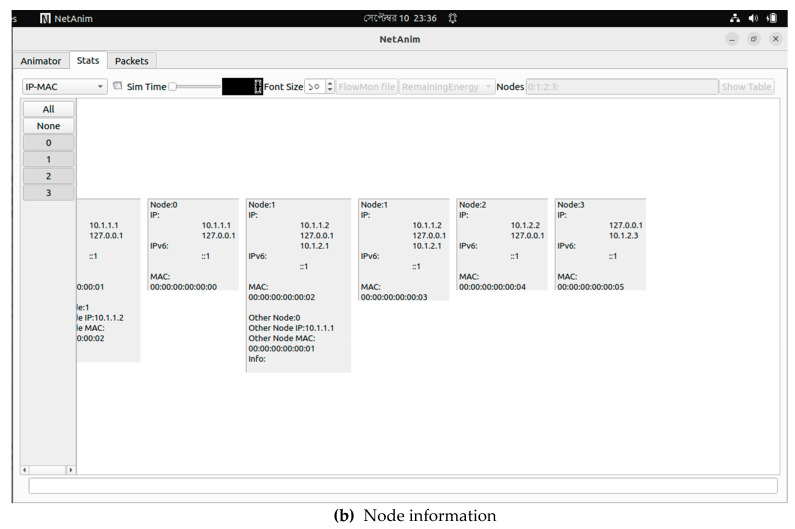
NS3 simulation from HMS to FTP and IoT. (**a**) Packet data passing; (**b**) Node information.

In part (ii), packets are sent from HMS to FTP and from HMS to IoT (Figure 12). This simulation utilized various nodes in this case, with node 0 serving as the source, node 1 serving as the HMS, node 2 serving as the FTP server, and node 3 serving as the IoT server. Table 3, Table 4, and Table 5 emerged from those simulations.

Image transfer response time was based on NS3 for performance evaluation, and the proposed model used three metrics: throughput, packet receipt rate and received packet size. This study calculated request response time based on packet size and bandwidth to trace the data transfer protocol from source to destination on the IoT server.

The outcomes are presented in Table 3, Table 4 and Table 5, where the data signify the request and response results depending on bandwidth and packet size. Furthermore, Table 3 and Table 4 indicate the reaction time when packet data are sent from ULSM to HMS and from HMS to FTP server, respectively. Based on the bandwidth and packet size, this study computed the response times.

**Figure 13:**
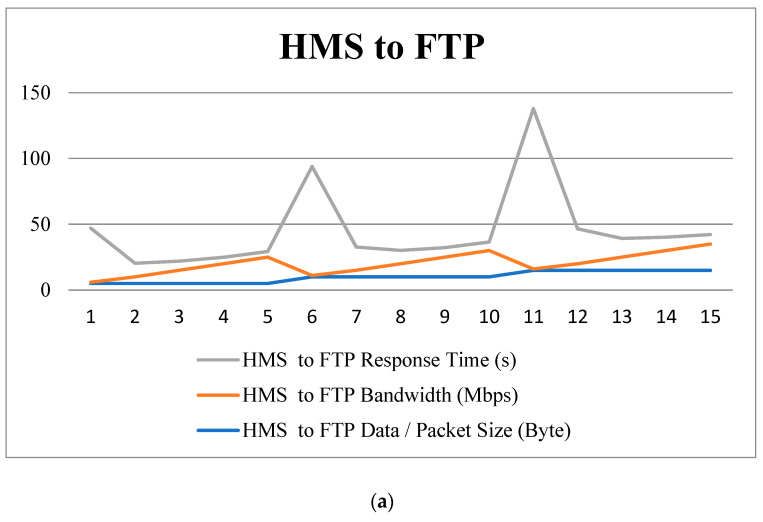

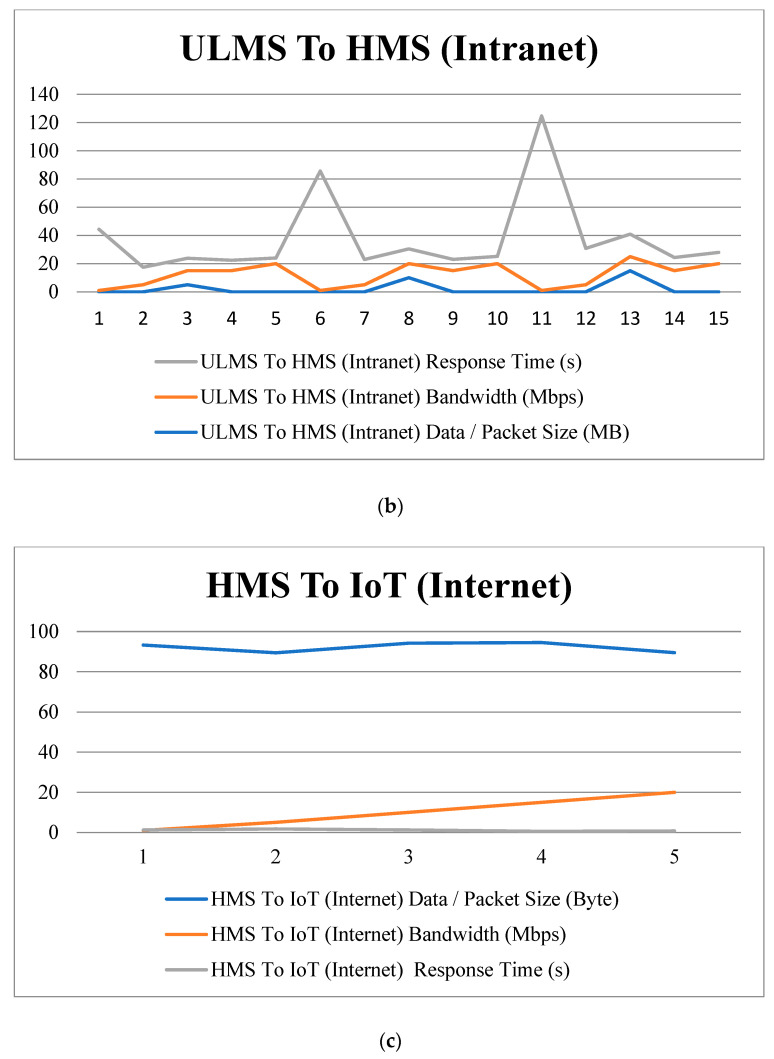
Line chart diagram based on the response time (**a**–**c**).

The data shown in Table 3 and Table 4 are displayed in the line charts in Figure 13, which implies that the response time and the package time increases. Additionally, when bandwidth increases, response time decreases. Again, Table 5 shows the response time from the HMS to the IoT server. From this outcome, we can see that response time is at a minimum below 3 s and, in line with that, the response time in Figure 13c is almost constant and not dependent on bandwidth because of the minimum packet size. To decrease the response time, this model has transferred telemetry data and an encrypted image link to the IoT server where encrypted image links are below 100 bytes.

Our proposed method is compared with others of the best health monitoring methods on the following criteria: (i) what types of data they are using, (ii) the machine they are collecting data from, (iii) if there is any machine-to-machine based communication, (iv) whether it supports real time monitoring, and (v) is there any IoT dashboard for displaying patient diagnostic information. Using those criteria, the proposed method is compared with other existing methods in Table 6.

With the use of the internet, we may access any LIS data from anywhere. In the IoT server, we can customize the dashboard or device access by sharing patient data in a variety of ways, including public, private, and personalized ways. Additionally, we have computed data source links using encryption, transmitted patient information, sent time data, and sent total data over time for the particular criterion. The results are based on how quickly the data are supplied to the IoT server, and the total number of images transmitted each minute. When we compare the criteria, we can see that the amount of data exchanged is dependent on time and is secure. This data line is directly connected to the ultrasonic machine.

A server-side module is used as the secure ultrasound real-time data channel and controls how ultrasound frame data are sent from the server to the client [20]. This data line connects directly to the ultrasonic machine, while parameters are managed via the secure real-time control route. Furthermore, the authors of one article used the LoRaWAN protocol for data transfer, whereas we merely produced byte data via URL encryption on the IoT server, which resulted in a long data response time on the IoT server [21]. Another article on sensor utilization used a smart healthcare system to collect data from the hospital environment by monitoring fundamental patient health indicators such as heart rate and temperature, whereas we use small packet data for displaying and generating large data with such health indicators and image sources [22]. Another study we encountered gave a process for creating professional, easy-to-understand visualizations of the data for seamless integration into clinical practice, whereas we have successfully developed a request-response-based data transfer and thus were able to reduce the data collision rate to a tolerable level [23]. In another piece of research, they attempted to adapt a system in an automated manner to follow the condition of patients when physicians are absent by employing SHS technology, which ensures that therapy is administered to a person in the absence of a doctor [24,25], which is quite dangerous. However, according to our system, patients do not need to rely on such risky treatment; they can easily send their issues in a request, and the system will store those data in a dashboard where doctors can monitor their registered patients’ issues and provide feedback on them from anywhere and at any time. The main hardware components are all based on the Raspberry Pi 3 Model B [26], and it enhances overall healthcare delivery by allowing patients and clinicians to interact in a variety of ways. Additionally, we have used only RSPI3 and large packet data as encrypted image links over FTP for displaying data in the IoT server, which consumes less time.

In contrast to the other studies above, our system overcame the large data transfer issue and displayed the image data, which may be a large amount of data. The response was returned on time and securely. Now we will discuss the conclusions of our proposed method and focus on whether it is the best method and if it is suitable for an IoT server.

## 4. Conclusions

Technology is constantly improving around the world, which is how IoT is becoming a more practical form of technology. Every industry is becoming more advanced because of IoT. The healthcare industry has also embraced IoT, and this paper is a straightforward reflection of how IoT may benefit the industry. Because many people may not have the opportunity to visit world-class healthcare experts, we created an IoT-based Ultrasound Image Monitoring System that allows healthcare experts or doctors to easily monitor and diagnose any patient from anywhere in the world, thanks to the benefits of cloud-based IoT systems. Our proposed method is applied over IoT from a hospital database, so it has some limitations e.g., for large data transfers it takes more time in real-time than the simulation, and the response time depends on the internet bandwidth.

In this approach, both doctors and patients will be able to save time and avoid superfluous costs such as making appointments with doctors, transportation fees, and other costs just by using our system properly. We hope that the technology we developed, as well as related research, will eventually bring about progress in the healthcare industry. Further modifications and improvements to the system will improve the overall service of the healthcare business around the world.

## Figures and Tables

**Table 1:** Thingsboard configuration summary (based on Figure 10).

Figure	Type	Feature List	Description
(a) Thingsboard Dashboard (Main)	Widget	Rule Chains	Signifies the data type, whether they are telemetry data or patient data
		Customer Management	Assign/Monitor customer roles in dashboard
		Assets	The type of device that will be directly connected to the IoT server i.e., ULSM, RSPI3
		Devices	Shows the list of device type and connections
		Device Profiles	
		OTA Updates	Installs and deploys system updates over-the-air (OTA) to devices
(a) Ultrasound Machine-based Dashboard	Summary Table	Action Buttons (View, Edit, Download, Share, Delete)	Organize and control the data
(c) Custom Widget for image display	Source Code	Custom Modification	Makes sure to decrypt the generated encrypted link by using RSA private key to display patient image data
(d) User-Based Dashboard Assign	Popup Module	User Roles	Data entry for customer info and role assignment

**Table 2:** Image display in IoT server by category.

Image Category (2D) Total Image per 30 s	Data Size	Network	Delay Minimum (s)	Display Medical Image Correctly (s)	Patient Information Found (s)
5 > image	Image < 1 MB	2G	15	10	5
5 < image < 10	1 MB < Image < 3 MB	3G	10	5	3
15 < image < 20	3 MB < Image < 5 MB	4G (LTE)	5	2	2
Image > 20	Image > 5 MB	Broadband above 5 Mbps	3	2	2

**Table 3:** ULSM to HMS (Intranet).

Data/Packet Size (MB)	Bandwidth (Mbps)	Response Time (s)
5	1	43.3442083
	5	12.40370939
	10	8.816277328
	15	7.366890492
	20	3.967757723
10	1	84.60675146
	5	17.92090079
	10	10.41828778
	15	8.005866191
	20	5.078943865
15	1	123.7062426
	5	25.7373848
	10	15.89119732
	15	9.30444808
	20	7.922080678

**Table 4:** HMS To FTP (Internet).

Data/Packet Size (MB)	Bandwidth (Mbps)	(S) Response Time (s)
5	1	41.64960943
	5	10.92773353
	10	5.634109647
	15	3.674797321
	20	4.611541816
10	1	81.17746564
	5	17.22091601
	10	10.01965412
	15	8.12423431
	20	6.908335693
15	1	122.5305769
	5	25.36579175
	10	14.6547472
	15	9.84765541
	20	7.490648534

**Table 5:** HMS To IoT (Internet).

Data/Packet Size (Byte)	Bandwidth (Mbps)	(S) Response Time (s)
93.2324224	1	1.252205553
89.36158621	5	1.726590514
94.16620009	10	1.217828047
94.49036105	15	0.5540504
89.44453449	20	0.738210043

**Table 6:** Comparison with existing methods.

Study	Data Type	Machine type	M to M Communication	Real time Monitoring	IoT Dashboard
[6]	Video	Ultrasound	No	Yes	No
[13]	Text	Sensors	No	No	No
[15]	Text	Sensors	Yes	No	No
[17]	Text	Sensors	Yes	Yes	No
[22]	Text	Sensors	Yes	Yes	Yes
Proposed Method	Text, Image	Ultrasound, MRI, CT	Yes	Yes	Yes

## Appendix A

**Table A1:** Connection message.

ARCHITECT-Initiated Connection Test:
MSH|^~\&|ARCHITECT|12345|||20091215153540+0000||NMD^N02^NMD_N02|d36bb8b2-8297-4372-be8a-87f38a86e739|P|2.5.1||||||UNICODE UTF-8SFT|ADD|8.0|ARCHITECT|”“ NST|N
**The following message is an example of an Acknowledgement to ARCHITECT initiated Connection Test:**
MSH|^~\&|||||20091215153541||ACK^N02^ACK|Ad36bb8b2-8297-4372-be8a-87f38a86e739|P|2.5.1||||||UNICODE UTF-8 MSA|AA|d36bb8b2-8297-4372-be8a-87f38a86e739

**Table A2:** Order message.

Order Message
<SB> MSH|^~\&|Machine Name |Machine Model| Source | Destination| Date Time||Order Code | Machine Model |P|2.3.1||||||UNI CODE PID| Patient Type|Patient Name|Patient ID|Sample ID|Test Type||Patient Date of Birth| Patient Gender<CR> ORC|1|Requist ID|Sample ID|MachineName^Machine Model||Sample Time| Start Time||||Test_Code |||||Li||<CR> ORC|2|Requist ID|Sample ID|MachineName^Machine Model||Sample Time| Start Time||||Test_Code |||||Li||<CR> ……. ORC|ORDER_SERIAL|Requist_ID|Sample ID|MachineName^Machine Model||Sample Time| Start Time||||Test_Code |||||Li||<CR> <EB><CR>

**Table A3:** Result message.

Result Message
<SB> MSH|^~\&|Machine Name |Machine Model| Source | Destination| Date Time||Order Code | Machine Model |P|2.3.1||||||UNI CODE PID| Patient Type|Patient Name|Patient ID|Sample ID|Test Type||Patient Date of Birth| Patient Gender<CR> OBR|1|Requist ID|Sample ID|MachineName^Machine Model||Sample Time| Start Time||||||Test Name || |Li||011<CR> OBX|1|ED|IMG1|Image1|Machine Model^Image^JPEG^Base64^……Base64_Code||||||F|||Item Result Time|011|Doctor Name|<CR> <EB> <CR>

**Table A4:** Acknowledgement message.

The Following Message Is an Example of an Acknowledgement to ARCHITECT Initiated Connection Test:
MSH|^~\&|||||20091215153541||ACK^N02^ACK|Ad36bb8b2-8297-4372-be8a-87f38a86e739|P|2.5.1||||||UNICODE UTF-8 MSA|AA|d36bb8b2-8297-4372-be8a-87f38a86e739

## Appendix B

**Table A5:** Private key.

Private Key
MIIBVQIBADANBgkqhkiG9w0BAQEFAASCAT8wggE7AgEAAkEAnXeCBmYdiea+ DFVtFIPOEZYGdnozXJhSyMnhynYScbk1rVGyZYcWHStjRlmmra2h1Y7APmYqKljcnUgei+GzewIDAQABAkArkYZraNOhdTN+ TCCbPYDFwuHU5CjT5N166sjLcPHXfvyFWu7I3SVHw3h1jOYdsMF4pJWGrq65KWQiWpbKXgRhAiEA381fUq9LaBxevvOwRaVGKeszA2jxXd5qfrXrB31awusCIQC0HwFLZihHl9YVaDNUbmwGtmO52eaDFVXnQq6iD/ENsQIhAKcTzRwPXb4ln93yUyBLWGwm+ HiNOdQYHWznJsT6om1tAiBB8/xoXF6xYFJ+ gioRZ2Fcz9oSSkxSgTR0OoFxS/8K8QIhAKjeIKNR3zUsPDj4JK/XVlFXD5/vV0eI8gCO4x1dLPJl

**Table A6:** Public key.

Public Key
MFwwDQYJKoZIhvcNAQEBBQADSwAwSAJBAJ13ggZmHYnmvgxVbRSDzhGWBnZ6M1yYUsjJ4cp2EnG5Na1RsmWHFh0rY0ZZpq2todWOwD5mKipY3J1IHovhs3sCAwEAAQ==

**Table A7:** Message to be encrypted.

Message to Be Encrypted
http://FTP_DOMAIN_NAME/PATH/UUID.IMAGE_EXTENTION

**Table A8:** Output/message to decrypt: [Encrypted Output on Base64].

Output/Message to be Decrypt: [Encrypted Output on Base64]
XHcVipE1xGw7291Kj9jJksm5xMbzQDxYhWb/IPXgyC3bjEEKzCPZr60ChsoPN2E/+HKD3i9/fIb5VmrJRUrt7w==

**Table A9:** Decrypted output.

Decrypted Output
shttp://FTP_DOMAIN_NAME/PATH/UUID.IMAGE_EXTENTION
